# Testing a hypothesis of unidirectional hybridization in plants: Observations on *Sonneratia, Bruguiera *and *Ligularia*

**DOI:** 10.1186/1471-2148-8-149

**Published:** 2008-05-16

**Authors:** Renchao Zhou, Xun Gong, David Boufford, Chung-I Wu, Suhua Shi

**Affiliations:** 1 State Key Laboratory of Biocontrol and Key Laboratory of Gene Engineering of the Ministry of Education, Sun Yat-Sen University, Guangzhou 510275, PR China; 2 Kunming Institute of Botany, Chinese Academy of Sciences, Kunming 650204, PR China; 3 Harvard University Herbaria, 22 Divinity Ave, Cambridge, Massachusetts 02138, USA; 4 Department of Ecology and Evolution, University of Chicago, Chicago, Illinois 60637, USA

## Abstract

**Background:**

When natural hybridization occurs at sites where the hybridizing species differ in abundance, the pollen load delivered to the rare species should be predominantly from the common species. Previous authors have therefore proposed a hypothesis on the direction of hybridization: interspecific hybrids are more likely to have the female parent from the rare species and the male parent from the common species. We wish to test this hypothesis using data of plant hybridizations both from our own experimentation and from the literature.

**Results:**

By examining the maternally inherited chloroplast DNA of 6 cases of F1 hybridization from four genera of plants, we infer unidirectional hybridization in most cases. In all 5 cases where the relative abundance of the parental species deviates from parity, however, the direction is predominantly in the direction opposite of the prediction based strictly on numerical abundance.

**Conclusion:**

Our results show that the observed direction of hybridization is almost always opposite of the predicted direction based on the relative abundance of the hybridizing species. Several alternative hypotheses, including unidirectional postmating isolation and reinforcement of premating isolation, were discussed.

## Background

Natural hybridization is a relatively common phenomenon in both plants and animals [1]. Patterns of interspecific hybridization are of great interest to many biologists, because they may provide insights into the mechanisms and evolution of reproductive barriers. One important aspect of the patterns involves the direction of hybridization. Numerous observations have shown that barriers to cross pollination are often asymmetric in plants [2-6]. Many hypotheses have been proposed for the asymmetry. Most concern the specific mechanisms by which the asymmetry is achieved. For example, some cytological, physiological and ecological mechanisms, such as ploidy level [4], breeding system [7] and unidirectional nuclear-cytoplasmic incompatibility [2,5], may account for the asymmetrical hybridization in specific cases. Here, we shall consider general hypotheses that i) do not invoke the specific reproductive biology of the plants in question, and ii) can account for hybridization patterns that depend on the relative species abundance in the hybrid zone.

One such hypothesis posits that hybridization tends to be unidirectional at sites where one species is rare, because the pollen delivered to the rare species would consist mainly of pollen of the common species [8-10]. Under such a condition, the rare species is usually the maternal parent of the hybrids [3,10]. A similar prediction has been made in animals as well [11].

In this study, we wish to test this prediction using data from four hybrid crosses in *Sonneratia*, *Bruguiera *and *Ligularia*. *Sonneratia *and *Bruguiera *are two genera of mangroves, and *Ligularia *is a genus of alpine and open meadow plants. All the individuals from the four interspecific crosses had been identified as simple F1s without further backcrossing by AFLP or ISSR markers [12-14]. Two additional examples from another genus of mangroves, *Rhizophora*, will be included in the discussion.

Even such a simple test requires information that cannot be gleaned readily from the literature. Additional experimental work, albeit straightforward, is often necessary. To test this hypothesis, data collection must meet the following three criteria: 1) the hybrids are F1s, rather than advanced hybrids (for such a confirmation, see [13]); 2) the relative abundance of the parental species has been surveyed and known to be highly skewed; and 3) the direction of hybridization has been determined. Although many examples of natural hybridization have been reported, few cases provide all the necessary information. The six cases reported here had to be verified experimentally.

Because chloroplast DNA is usually maternally transmitted in angiosperms [15,16], it has been widely used to identify the maternal parent of the hybrids [17-22]. In this study, we first wanted to determine if chloroplast DNA was also maternally inherited in the three genera using cytological experiments. Once maternal inheritance was confirmed, we sequenced the *trn*L/F (and more for *Bruguiera*) regions of the chloroplast DNA from the hybrids and their parents to determine the direction of hybridization. We chose these regions because noncoding chloroplast DNA sequences like *trn*L/F can often be used to distinguish closely related species [23,24]. We also conducted field surveys to assess the composition of the parental species in the hybrid zones of *Sonneratia *and *Bruguiera*. Our goal was to address the following questions: 1) Is hybridization unidirectional in these examples? 2) If so, in what direction did the hybridization happen? and 3) Could the existing hypothesis explain our observations?

## Results

### Maternal inheritance of chloroplast DNA in Sonneratia and Bruguiera

**Figure 1:**
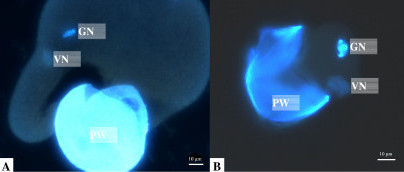
DAPI-stained epifluorescence microphotographs showing squashed pollen grains of (A) *Sonneratia alba *and (B) *Bruguiera gymnorrhiza*. GN, generative nucleus; VN, vegetative nucleus; PW, pollen wall.

We used DAPI (4',6-diamidino-2-phenylindole)-staining fluorescence microscopy to determine if chloroplast DNA is maternally inherited in a certain plant (see Methods). We chose one species from each genus, *S. alba *from *Sonneratia *and *B. gymnorrhiza *from *Bruguiera*, to examine the mode of inheritance. In our study, no punctated fluorescence corresponding to plastid DNA aggregates was observed in mature pollen grains of *Sonneratia alba *and *Bruguiera gymnorrhiza *(Fig. 1), indicating the mode of maternal chloroplast DNA inheritance in these species. Maternal inheritance of chloroplast DNA in *Ligularia *has been identified before [25], so chloroplast DNAs are maternally inherited in all three of these genera.

### The direction of hybridization in Sonneratia

The chloroplast *trn*L/F regions in *Sonneratia *exhibited limited variation. The sequences of the chloroplast *trn*L/F region generated from the five taxa in *Sonneratia *were consistently 1000 bp in length. Sequences of different accessions from each taxon were identical. In total, there were four nucleotide substitutions among the three parental species (Table 1). Three nucleotide substitutions differentiate *S. caseolaris *from *S. alba *and *S. ovata*. Two nucleotide substitutions exist between *S. alba *and *S. ovata*. The 12 accessions of *S*. × *gulngai *(hybrid between *S. alba *and *S. caseolaris*) and 6 accessions of *S*. × *hainanensis *(hybrid between *S. alba *and *S. ovata*) we sampled in the hybrid zones had the same *trn*L/F sequence as *S. alba*.

**Table 1:** Chloroplast genotypes in hybrids and parental species of *Sonneratia, Bruguiera, Ligularia *and *Rhizophora*

Genera (chloroplast markes used)	Hybrids (Number of individuals sampled)	Parental species (Number of individuals sampled)	Hybrid genotypes	Source
*Sonneratia *(*trn*L/F)	*S*. × *gulngai *(12)	*Sa *(15) and *Sc *(12)	Same as *Sa*	This study
	*S*. × *hainanensis *(6)	*Sa *(15) and *So *(10)	Same as *Sa*	
*Bruguiera *(*trn*S-*trn*G/*psb*B-*psb*F)	*B*. × *rhynchopetala *(34)	*Bg *(20) and *Bs *(20)	Same as *Bg *(5) Same as *Bs *(29)	This study
*Ligularia *(*trn*L/F)	*L. paradoxa *× *duciformis *(16)	*Lp *(20) and *Ld *(20)	Same as *Lp*	This study
*Rhizophora *(*trn*S-*trn*G/*trn*H-*rpl*2)	*R*. × *annamalayana *(3)	*Rm *(3) and *Ra *(3)	Same as *Rm*	Lo et al. 2003 [26]
	*R. × lamarkii *(7)	*Rs *(7) and *Ra *(5)	Same as *Rs*	

Sa, Sonneratia alba; Sc, S. caseolaris; So, S. ovata; Bg, Bruguiera gymnorrhiza; Bs, B. sexangula; Lp, Ligularia paradoxa; Ld, L. duciformis; Rm, Rhizophora mucronata; Ra, R. apiculata; Rs, R. stylosa.

During our field survey, we found *Sonneratia alba *and *S. caseolaris *to be roughly equally abundant (between 1:1.5 and 1.5:1) either in the exact zone of contact or in adjacent areas (Table 2). In the hybrid zone of *S. alba *and *S. ovata*, their relative abundance is highly skewed toward *S. alba*, with a ratio of approximately 10:1 for *S. alba*: *S. ovata *(Table 2).

**Table 2:** Relative abundance of parental species in the contact zone and the direction of hybridization in 6 cases from 4 genera

Hybrid (Location)	Relative abundance of parental species	Direction of hybridization
*Sonneratia* × *gulngai* (Qionghai, Hainan) (Wenchang, Hainan)	*S. alba: S. caseolaris* ~1:1 (71:97; 664:573) ~1:1 (83:112; 556:477)	(equally common)
*S*. × *hainanensis *(Wenchang, Hainan)	*S. alba*: *S. ovata *~10:1 (340:37)	C♀ × R♂
*Bruguiera *× *rhynchopetala *(Haikou, Hainan)	*B. sexangula*: *B. gymnorrhiza *~2:1 (98:53)	C♀ × R♂ (29) R♀ × C♂ (5)
*Ligularia paradoxa *× *duciformis *(Maoniushan, Yunnan)	*L. paradoxa*: *L. duciformis *~5:1	C♀ × R♂
*Rhizophora *× *annamalayana *(West coast, Sri Lanka)	*R. mucronata*: *R. apiculata *~100:1	C♀ × R♂
*R*. × *lamarkii *(Daintree River and Shoalwater Bay, Australia)	*R. stylosa*: *R. apiculata *~100:1	C♀ × R♂

**Note: **For *Sonneratia *× *gulngai*, the hybrid zones both in Qionghai and in Wenchang are about 1 km long. We recorded the number of individuals of the parental species both within the zone of hybridization (the former) and in the neighboring 3 km-long zone (the latter). The latter zone is composed solely of parental species. For *S*. × *hainanensis*, we counted the number of individuals of *S. alba *only around the range of *S. ovata *and *S*. × *hainanensis*. Other cases are restricted the exact zones of hybridization.

In both instances of interspecific crosses, hybridization was unidirectional and *S. alba *was the maternal parent. Because the parental species of *S*. × *gulngai *are roughly equally abundant, the hypothesis based on relative abundance makes no prediction for direction of hybridization. In *S*. × *hainanensis*, the direction of hybridization was opposite the prediction of the hypothesis.

### The direction of hybridization in Bruguiera

Although there is remarkable morphological difference between *Bruguiera gymnorrhiza *and *B. sexangula*, variation in the regions of the chloroplast genome is scarce. Among the eight intergenic regions sampled, only the *psb*B-*psb*F and *trn*S-*trn*G intergenic spacers exhibited variation (Table 1) and were thus chosen as diagnostic markers in the subsequent analysis. The amplified *psb*B-*psb*F intergenic spacers of *B. gymnorrhiza *and *B. sexangula *differ by about 50 bp in length (*B. gymnorrhiza*: about 900 bp and *B. sexangula*: about 950 bp). The amplified *trn*S-*trn*G intergenic spacers differ by a nucleotide substitution (site 197: A/C). No within-species variation was observed in the sequences of the two regions for all 20 individuals of *B. gymnorrhiza *and all 20 individuals of *B. sexangula *examined.

Among the 34 samples of *B*. × *rhynchopetala *examined in this study, the majority (29) had the *B. sexangula *chloroplast genotype, while a minority (5) possessed the *B. gymnorrhiza *chloroplast genotype. Our survey showed that the number of adult individuals of *B. sexangula *is about twice that of *B. gymnorrhiza *(98:53) in the hybrid zone. The expected direction of hybridization should thus be predominantly *B. gymnorrhiza *(♀) × *B. sexangula *(♂). However, our observation showed the opposite, as the direction was predominantly *B. sexangula *(♀) × *B. gymnorrhiza *(♂).

### The direction of hybridization in Ligularia

The sequences of the chloroplast *trn*L/F region generated from the *Ligularia *hybrids and their parents, *L. paradoxa *and *L. duciformis*, were consistently 852 bp in length. No within-species sequence variation was observed for the parental species. There were three nucleotide substitutions between the parental species (Table 1). All 16 accessions of the hybrids had sequences identical to the sequences of *L. paradoxa*. The number of individuals of *L. paradoxa *was about five times that of *L. duciformis *in their zone of contact (X. Gong, Y. Pan and S. Shi, unpublished data). It is therefore expected by the hypothesis that the rarer species, *L. duciformis*, should be the maternal parent. Our observations showed the opposite.

## Discussion and Conclusion

Asymmetry in the direction of interspecific hybridization was observed in all three genera. In our samples, the crosses were unidirectional in *Sonneratia *and *Ligularia*, and strongly asymmetric in *Bruguiera*. The maternal parents of these hybrids were predominantly from the common species.

Two additional examples of unidirectional hybridization have been reported from another genus of mangroves, *Rhizophora *– *R*. × *annamalayana *(*R. mucronata *× *R. apiculata*) in Sri Lanka, and *R*. × *lamarkii *(*R. stylosa *× *R. apiculata*) along the Daintree River and at Shoalwater Bay in eastern Australia [26]. The ratio of the number of individuals of the parental species was about 100:1 in both instances (N. Duke, personal communication) and the hybrid individuals have the same *trn*H-*rpl*2 and *trn*S-*trn*G sequences as the common species – *R. mucronata *in Sri Lanka and *R. stylosa *in eastern Australia.

In our study, the direction of hybridization is opposite of expected in all five cases where the relative abundance of parental species deviates from 1. We assume that pollen quantity is correlated with the number of individual trees, as is generally true between species that have similar floral structures and are capable of hybridizing. Our results do not support the prediction that the minority species usually acts as the maternal parent.

To explain our observations, we separately consider postmating and premating reproductive isolation. For the former, we discount the distinction between R♀ × C♂ and C♀ × R♂ crosses (R and C refer to Rare and Common species in the hybrid zone, respectively) in our observations. We note that the only case of bi-directional hybridization in Table 2 happens to be the one with the lowest bias in parental abundance (in *Bruguiera*). Whether this relative abundance is considered will have bearings on the interpretation of the data.

### Postmating isolating mechanisms

There are several postmating isolating mechanisms known to cause unidirectional hybridization. These mechanisms, which include nuclear-cytoplasmic interactions, X-autosome interactions, maternal effects and asymmetric incompatibilities, have been clearly summarized by Turelli and Moyle [27]. On the list, we may add ploidy level. The genomes of organelles may contribute genetic information that critically affects the survivorship of their progeny, so hybrids of reciprocal crosses may differ in survivability [2,5,19]. In angiosperms, it has been reported that pollen from self-incompatible species is able to fertilize ovules from self-compatible species, but not the reverse, a condition referred to as the "SI × SC rule" [1,7,28,29]. Different ploidy levels between hybridizing species could also contribute to asymmetrical hybridization, as larger cell size associated with polyploidy might influence the physical attributes of the motility of the sperm [4].

Among these mechanisms, nuclear-cytoplasmic incompatibility may be a most widespread one. F1's from the two reciprocal crosses are genetically identical in the nuclear genes. This is true for the homogametic hybrids in species with sex chromosomes or for both sexes in other species. Because the number of genes on the organelle genomes is so much smaller than that of nuclear genes, postmating isolation is often expected to have comparable strength in both directions. This is indeed the case in animal hybridizations (see [30] for references). In plants, the sizes of organelle genomes are much larger than those of animals and, hence, asymmetry in postmating isolation may be much more common. The other mechanisms may operate in subsets of plant taxa.

Turelli and Moyle [27] gave the theoretical conditions when these various mechanisms may play a significant role. Since these conditions are often not known, an alternative empirical approach is to tally the number of cases of observed asymmetric postmating isolation. Tiffin et al. [5] have provided such an extensive survey and concluded that asymmetric postmating isolation is common in angiosperms.

In the survey of Tiffin et al. [5], modest but significant asymmetry is prevalent. If the viability of F1s in one direction is, say, 80% of that in the other direction, the difference of 20% was often significant. In contrast, such moderate asymmetry should have been observed as bidirectional hybridization in our study. For the purpose of our study, strong asymmetry and unidirectional isolation are most crucial. We therefore quantify the strength of asymmetry in a subset of samples that were also included in Tiffin et al. [5]. We notice that the distribution of cases with symmetry, moderate asymmetry and strong asymmetry in postmating isolation [see Additional file 1] is roughly 50%, 25% and 25% across the 6 genera. The results, shown in Table S1, suggest that the distribution may be applicable to each genus surveyed.

If we apply the 50%:25%:25% distribution to the genera of Table 2, there would be a 25% chance for each case of postmating isolation to be nearly unidirectional. Thus, without taking into account the relative abundance of the two parental species in the hybrid zone, one could conclude unidirectional postmating isolation to be a reasonable explanation for the pattern of Table 2. However, summing over the five crosses, the ratio of [C♀ × R♂]: [R♀ × C♂] crosses is 61:5. As the ratio is expected to be much less than 1, which is the basis of the hypothesis motivating this study, the full pattern of Table 2 may require additional considerations.

### Premating isolating mechanisms

While the influence of postmating mechanisms on the pattern of Table 2 cannot be ignored, none of these mechanisms is likely to depend on the relative abundance of parental species in the hybrid zone. In contrast, the relative abundance may be important in premating isolation. We therefore suggest reinforcement of premating isolation to be a reasonable hypothesis for further testing. Although Table 2 includes all cases that could be found so far, it is very much limited in scope. We propose the hypothesis also to emphasize the need for further data collection and more comprehensive testing.

The reinforcement hypothesis posits that natural selection can strengthen premating isolation between sympatric taxa as a response to the existing postmating isolation [31]. If two species have evolved postmating isolation and have become sympatric for some time, the crossing barriers might be reinforced by mechanisms that prevent the common R♀ × C♂ crosses. In contrast, the rarer C♀ × R♂ cross has experienced relatively weak selective pressure and might have escaped reinforcement. Under the reinforcement hypothesis, hybrids, when they do form, may come predominantly from the direction of C♀ × R♂. The arguments and hypothesis presented above are of course not new. They have been presented in one form or another in the context of hybrid zone or in the discussion of interspecific character displacement [32,33].

There are several caveats in inferring reinforcement from the preponderance of C♀ × R♂ hybridizations, if this preponderance is indeed the trend. Several ancillary observations will be needed.

First, the species should have some forms of postmating isolation. One such example in Table 2 is the hand-crossing experiments with *S. alba *as the pollen donor and *S. ovata *as the maternal parent. In such a cross, the ovaries did not expand and no fruits or seeds were formed (C. Zhong, personal communication). Furthermore, the three hybrids in *Sonneratia *and *Ligularia *have much reduced fitness in comparison with their parental species. The proportion of sterile pollen in either *S*. × *gulngai *(95.62%) or *S*. × *hainanensis *(54.43%) is much higher than in the parental species, *S. alba *(8.76%), *S. caseolaris *(5.68%), and *S. ovata *(3.25%) [34]. Even more extreme, the germination rate of the seeds from the *Ligularia *hybrid is zero [14].

Second, the hybridizing species should have been in sympatry long enough for reinforcement to evolve. In contrast to the situations in our study, many hybridization events result from habitat disturbances associated with human activities [35,36] and the contact between the parental species has been recent. In these situations, either the hybrid zone is too young or the degree of postzygotic isolation is too weak. Hence, reinforcement may not have had time to evolve and hybridizations often follow the numerical expectation. In our examples, the hybrid zones for *Sonneratia*, *Bruguiera *and *Ligularia *are unlikely to be recent. The fossil pollen record shows that *Sonneratia *and *Bruguiera *have existed on Hainan since the Pleistocene [37]. The hybrid zone of *Ligularia *is in a region of little human disturbance. Thus there should have been sufficient time for reinforcement to operate within these hybrid zones.

Conclusive evidence in support of reinforcement in plants is hard to come by [38,39]. Our suggestion of reinforcement is based on indirect inference. Direct tests entail empirical manipulations that bring together allopatric individuals from different species [40,41]. Such manipulations may not be feasible in many taxa, and surveys of hybridization patterns in nature are another option. If interspecific hybridization is consistently in the direction of C♀ × R♂, then reinforcement could be of some importance in plants.

## Methods

### Plants studied

*Sonneratia *L.f. (Lythraceae *sensu lato*) is a genus of mangrove plants comprising four to six species and three interspecific hybrids [13,34,42-47]. The genus is widely distributed in the Indo-West Pacific (IWP) region. In China, *Sonneratia *occurs naturally only on Hainan Island, where it includes three indigenous species, *S. alba *J. Smith, *S. ovata *Backer, and *S. caseolaris *(L.) Engler. *Sonneratia alba*, a pioneering mangrove tree, has the widest distribution in Hainan, with a range from Sanya, Lingshui and Qionghai to Wenchang. *Sonneratia caseolaris *occurs in Wanning, Qionghai and Wenchang. *Sonneratia ovata *is restricted to Wenchang. The main differences in flowers among the three species lie in the absence/presence of petals and the color of the filaments. Both *S. alba *and *S. caseolaris *have inconspicuous petals, but they differ in the color of the petals and filaments, white in the former and red in the latter. *Sonneratia ovata *lacks petals and the filaments are white. The species of *Sonneratia *flower nearly throughout the year and are predominantly outcrossers, with bats and birds as the main pollinators [48,49]. There are two natural hybrids in the genus *Sonneratia *on Hainan Island, i.e., *S*. × *gulngai *(*S. alba *× *S. caseolaris*) and *S*. × *hainanensis *(*S. alba *× *S. ovata*) [13]. The two hybrids and their parental species are all diploids, with the same chromosome number of 2n = 22 [50].

*Bruguiera *Sav. (Rhizophoraceae), consisting of six species [48,51], is another genus of mangroves distributed in the Indo-West Pacific (IWP) region. Two species of *Bruguiera*, *B. gymnorrhiza *(L.) Sav. and *B. sexangula *(Lour.) Poir, have widely overlapping geographic ranges throughout the IWP region. The large, solitary, recurved flowers of both species of *Bruguiera *are considered to be bird-pollinated [48]. Because of the high failure ratio under conditions of autogamy [52], these two species of *Bruguiera *should have mating systems with high levels of outcrossing. As in the species of *Sonneratia*, they flower nearly throughout the year on Hainan. Their flowers resemble each other to a large extent and differ only in having the petal lobes either blunt or acute and in the presence or absence of filamentous appendages at their tips [48]. In China, they are sympatric in Dongzhai Harbor, Hainan, and produce the hybrid *Bruguiera *× *rhynchopetala *[12]. The three taxa of *Bruguiera *have the same chromosome number of 2n = 36 [50].

*Ligularia *Cass. (Asteraceae) is a genus distributed mainly in eastern Asia. It consists of about 130 species, among which 112 species occur in China [53]. A naturally-occurring hybrid between *Ligularia paradoxa *Hand.-Mazz. and *L. duciformis *Hand.-Mazz. from Maoniu Shan, northwestern Yunnan, China, has been identified based on morphological and cytological observations and ISSR markers [14]. The hybrid occurs in the area of overlap between the parental species in a coniferous forest, at an altitude of 4200 m. Both *L. duciformis *and *L. paradoxa *belong to Series *Retusae *in Section *Corybosae *[54] and have very similar flower structures. The main floral difference between them is in the color of the pappus. *Ligularia paradoxa *has a white pappus and *L. duciformis *has a purple pappus. They do, however, show remarkable differences in the shape of the leaves. *Ligularia paradoxa *and *L. duciformis *have deeply partite and entire leaves, respectively. *Ligularia duciformis *flowers from July to September, and *L. paradoxa *flowers from July to August, with an overlap of two months in their contact zones [14]. Bumblebees are the most frequent pollinators, promoting outcrossing in the two species. The *Ligularia *hybrid and its parental species have the same chromosome number of 2n = 58 [14].

### Examination of maternal inheritance of chloroplast DNA in Sonneratia and Bruguiera

When pollen cells of species known to exhibit paternal plastid transmission were stained with 4',6-diamidino-2-phenylindole (DAPI), punctated fluorescence corresponding to plastid DNA aggregates was invariably associated with mature generative or sperm cells [55]. Conversely, plastid DNA is absent in the generative cells or sperm cells of species known to display maternal inheritance. So DAPI (4',6-diamidino-2-phenylindole)-staining fluorescence microscopy can be used to determine if chloroplast DNA is maternally inherited in a certain plant. Since maternal chloroplast inheritance in *Ligularia *has been identified [25], here we examined the mode of chloroplast inheritance in *Sonneratia *and *Bruguiera*. We chose one species from each genus, *S. alba *from *Sonneratia *and *B. gymnorrhiza *from *Bruguiera*, to examine the mode.

Epifluorescence microscopy of chloroplast DNA was performed according to the method of Zhang et al. [25]. Mature pollen grains of *Sonneratioa alba *and *Bruguiera gymnorrhiza *were collected from Dongzhai Harbor Mangrove Nature Reserve, Hainan. Pollen grains were placed on a glass slide and immersed in a drop of 3% glutaraldehyde and 1 μg ml^-1 ^DAPI in Tan buffer [56]. The pollen grains were covered with a cover slip, and then squashed by pressing the cover slip against the slide. The samples were examined after 10 min with an Olympus BH-2 epifluorescence microscope.

### Ecological surveys in Sonneratia and Bruguiera hybrid zones

On Hainan, *Sonneratia alba *and *S. caseolaris *occur sympatrically at Qionghai and Wenchang, where hybrid zones are formed. *Sonneratia alba *and *S. ovata *coexist only at Wenchang. All three species on Hainan occur within estuaries. The physiological tolerance of each species to salinity determines its habitat. Both *S. alba *and *S. ovata *grow on the more salty seaward side of mangrove forests, but they differ in their intertidal locations; *S. alba *prefers the low intertidal zone, whereas *S. ovata *inhabits only the high intertidal zone. By contrast, *S. caseolaris *grows on the less salty inland side, and along streams. The hybrids form only in the narrow intermediate area between the ranges of the parental species, usually within a distance of less than 1 km. Our search for *S*. × *gulngai *was conducted along the estuary systems at Tanmen, Qionghai, and Qinglan, Wenchang. We focused on the areas where hybrids were found (about 1 km), with a 3 km extension in both directions towards the populations *S. alba *and *S. caseolaris*. For *S*. × *hainanensis*, our survey was confined to Qinglan, Wenchang, where we found 37 individuals of *S. ovata *and only 6 individuals of *S*. × *hainanensis *during three field surveys. We counted the number of individuals of *S. alba *only within the range of *S. ovata *and *S*. × *hainanensis*. During the surveys, the number of individuals of each parental species was recorded for all the adult trees.

In China, there are only two species in *Bruguiera*, *B. gymnorrhiza *and *B. sexangula*. *Bruguiera gymnorrhiza *usually inhabits the downstream and intermediate locations of estuary systems while *B. sexangula *inhabits the upstream and intermediate locations. Both taxa are usually located in mid to high intertidal zones of mangrove stands. A hybrid zone is formed at Dongzhai Harbor Mangrove Nature Reserve (20° 00'N and 110° 35'E), Haikou. We recorded the quantity of adult individuals of *B. gymnorrhiza *and *B. sexangula *within this hybrid zone.

### Plant collections

Three species of *Sonneratia*, *S. alba*, *S. caseolaris *and *S. ovata*, and two hybrids *S*. × *gulngai *and *S*. × *hainanensis *were sampled at Qionghai and Wenchang, Hainan. Table 1 shows the number of samples collected for each species.

In *Bruguiera*, 20 individuals of *B. gymnorrhiza*, 20 individuals of *B. sexangula*, and 34 hybrid individuals were sampled in the hybrid zone of Dongzhai Harbor Mangrove Nature Reserve.

The hybrids and parental species of *Ligularia *were sampled at Maoniu Shan, Yunnan. Fifty-six individuals (20 of *L. duciformis*, 20 of *L. paradoxa *and 16 hybrids) were sampled.

These hybrids were identified by their intermediate morphological characteristics in the field. Samples of each hybrid were collected across the whole hybrid zone, and intervals between samples were at least 15 m. For each sample from the three genera, leaves were collected and stored in sealed plastic bags with silica gel until DNA extraction. Voucher specimens were deposited in the herbarium of Sun Yat-Sen University (SYS). In all of these cases, morphological intermediacy was a very effective criterion for identifying hybrids. All hybrid individuals with morphological intermediacy in these three genera, albeit unmentioned in the original studies on *Bruguiera *[12] and *Ligularia *[14], were indeed F1s, as reasoned below.

Multi-locus molecular markers such as AFLP and ISSR can be used to assess the extent of interspecific hybridization. In our analysis, only monomorphic species-specific bands were recorded. As described in our previous paper [13], we designate the genetic contribution to the hybrids from either parental species as p and q (p + q = 1), respectively. In the F1 generation, p = 0.5 = q. With one backcross, p = 0.75 and q = 0.25. p and q can range between 0 and 1 for more complex scenarios. In the F2 generation, the expected proportion of bands in the hybrids that are homozygous for either parent is 0.25. Homozygosity for the allele from parent 1 means the absence of the band from parent 2. In the hybrids of more advanced generations, the proportion of homozygotes for alleles from parent 1, heterozygotes, and homozygotes for alleles from parent 2 would be, respectively, [p^2^(1-F)+pF]: [2pq(1-F)]: [q^2^(1-F)+qF], where F is the inbreeding coefficient between 0 and 1 [57]. Therefore, the total proportion of missing bands would be half the sum of the two proportions for homozygotes: [(1 - F)(p^2 ^+ q^2^)+ F]/2. The division by 2 is due to the fact that one of the two parental bands would be missing at each homozygous locus. The smallest number from Eq. (1) is 25% for F2's, when p = q = 0.5 and F = 0.

According to this criterion, it is expected that F1 hybrids would have all the parental species-specific bands, F2 hybrids would miss 25% of the species-specific bands of both parents, backcross one (BC1) hybrids will miss 50% of the species-specific bands of the non-recurrent parent, and hybrids of more advanced generations would lose over 25% of the species-specific bands of the parental species.

In *Sonneratia *× *gulngai *and *S*. × *hainanensi*s, the proportion of missing species-specific bands is 3.1% and 3.7%, respectively [13]. In *Bruguiera*, all 34 hybrid individuals collected possess 9 of 10 *B. gymnorrhiza*-specific bands and all 10 *B. sexangula*-specific bands [12]. In *Ligularia*, all 16 hybrid individuals collected have all 5 *L. paradoxa*-specific bands and all 15 *L. duciformis*-specific bands [14]. In each of the three genera, the total proportion of missing bands in all hybrid individuals is no more than 5%. Because nearly all markers from both species were present, putative hybrids appeared to be heterozygous for virtually all species-specific bands, so we can safely conclude that none of the hybrid individuals above can be anything other than F1 hybrids.

There is another possibility that some backcross hybrids may resemble either parent morphologically and may have escaped detection. Nevertheless, we have not found any sample that is morphologically one parental species but is molecularly a hybrid with some specific bands of the other parental species.

### DNA extraction, PCR amplification and sequencing

Total genomic DNAs were extracted from dried leaf tissues using the CTAB method [58]. The chloroplast *trn*L/F region was amplified using the universal primers trn-c and f [23]. Amplification conditions were as follows: 1 cycle at 94°C for 4 min; 28 cycles at 94°C for 45 s, at 58°C for 45 s, at 72°C for 2 min, followed by 1 cycle at 72°C for 8 min. The PCR products were purified by electrophoresis through a 1.2% agarose gel followed by use of an E.Z.N.A.^® ^Gel Extraction Kit (Omega). All the accessions for each taxon were subjected to chloroplast *trn*L/F sequencing. Sequencing was conducted with amplification primers in an ABI 3730 DNA automated sequencer with the BigDye Terminator Cycle Sequencing Kit (Applied Biosystems).

No sequence variation, however, was found in the *trn*L/F regions of the two species of *Bruguiera*. We then chose seven other regions of the chloroplast genome, namely, the *rpl20*-*rps12 *intergenic spacer, the *psb*B-*psb*F intergenic spacer, the *trn*S-*trn*G intergenic spacer, the *trn*H-*psb*A intergenic spacer, the *psb*B gene, the *rps*16-*rps*12 intergenic spacer, and the *atp*H-*atp*I intergenic spacer, to seek variation between the two species by using two individuals from each species. These regions were amplified using universal primers developed by Hamilton [59] and Grivet et al. [60]. We observed variation only in the *psb*B-*psb*F intergenic spacer and the *trn*S-*trn*G intergenic spacer. We therefore used these two spacers as diagnostic markers in subsequent experiments. PCR amplification and sequencing were conducted by using the same methods mentioned above. All sequences have been deposited in GenBank with accession numbers AY395722-AY395727, DQ865240-DQ865243 and DQ104429-DQ104431. The sequences were aligned and compared in SeqMan™ (DNASTAR).

## Authors' contributions

SS and RZ conceived and designed this study. RZ and XG collected materials and performed experiments. RZ and SS analyzed the data. RZ, DB, CIW and SS wrote the paper. All authors read and approved the final manuscript.

## Supplementary Material

Additional file 1 Number of reciprocal crosses that exhibited symmetry, moderate asymmetry and strong asymmetry in postmating isolation in six plant genera. These data describe the distribution of cases with symmetry, moderate asymmetry and strong asymmetry in postmating isolation of plants based on a literature survey. Click here for file
