# Supplementary material for: Testing a hypothesis of unidirectional hybridization in plants: Observations on Sonneratia, Bruguiera and Ligularia
Source: BMC Evol Biol. 2008 May 16;8:149. doi: 10.1186/1471-2148-8-149 (PMC2409324; doi:10.1186/1471-2148-8-149)
Supplement: Additional file 1 — Number of reciprocal crosses that exhibited symmetry, moderate asymmetry and strong asymmetry in postmating isolation in six plant genera. These data describe the distribution of cases with symmetry, moderate asymmetry and strong asymmetry in postmating isolation of plants based on a literature survey. [file 1471-2148-8-149-S1.doc]

**Additional file 1**

**Table S1** Number of reciprocal crosses that exhibited symmetry, moderate asymmetry and strong asymmetry in postmating isolation in six plant genera. Measures of postmating isolation include percentage of seed or fruit set and the proportion of seeds that are viable. We designate that symmetry, moderate asymmetry and strong asymmetry when the ratios in postmating isolation of two-way crosses are > 0.5, 0.5 ~ 0.1 and < 0.1, respectively. For some crosses where no seed or fruit set is formed, an arbitrarily small number 0.01 was used in place of 0.

| Genus | # of reciprocal crosses | # of symmetry | # of moderate asymmetry | # of strong asymmetry | Reference |
| --- | --- | --- | --- | --- | --- |
| *Paspalum* | 8 | 3 | 3 | 2 | 1 |
| *Phlox* | 31 | 20 | 7 | 4 | 2 |
| *Heuchera* | 29 | 20 | 0 | 9 | 3 |
| *Aphelandra* | 45 | 18 | 16 | 11 | 4 |
| *Guizotia* | 8 | 2 | 3 | 3 | 5 |
| *Ipomoea* | 11 | 6 | 3 | 2 | 6 |
| Total | 132 | 69 | 32 | 31 | ­－ |

**References**

[1] Quarin CL, Norrman GA: **Interspecific hybrids between 5 *Paspalum* species**. *Bot Gaz* 1990, 151: 366-369.

[2] Levin D: **The *Phlox pilosa* complex: Crossing and chromosome relationships.** *Brittonia* 1966, 18:143-162.

[3] Wells EF: **Interspecific hybridization in eastern North American *Heuchera* (Saxifragaceae)**. *Syst Bot* 1979, 4: 319-338.

[4] McDade LA, Lundberg JG: **A new tabular and diagrammatic method for displaying artificial hybridization data with an example from *Aphelandra* (Acanthaceae).** *Syst Bot* 1982, 7: 13-25.

[5] Dagne K: **Meiosis in interspecific hybrids and genomic interrelationships in *Guizotia* Cass. (Compositae).** *Hereditas* 1994, 121: 119-129.

[6] Diaz J, Schmiediche P, Austin DF: **Polygon of crossability between eleven species of *Ipomoea*: section *Batatas* (Convolvulaceae).** *Euphytica* 1996, 88: 189-200.
